# Antiproliferative Activity and Apoptosis Induction of Crude Extract and Fractions of *Avicennia Marina*

**Published:** 2013-11

**Authors:** Amir abbas Momtazi-borojeni, Mandana Behbahani, Hojjat Sadeghi-aliabadi

**Affiliations:** 1 Department of Biotechnology, Faculty of Advanced Sciences and Technologies, University of Isfahan, Isfahan, Iran; 2 Department of Pharmaceutical Chemistry, Faculty of Pharmacy and Pharmaceutical Sciences, Isfahan University of Medical Sciences, Isfahan, Iran

**Keywords:** Apoptosis, *Avicennia marina*, Cytotoxic activity, DNA fragmentation, MDA-MB 231 cell line

## Abstract

***Objective(s):*** Regarding the presence of many active biological constituents in Avicennia marina, the present investigation was carried out to study cytotoxic activity of crude methanol leave extract and column chromatographic fractions of A. marina against MDA-MB 231 cell line (human breast cancer cell) and HEK (Human embryonic kidney cell) line.

***Materials and Methods:*** The anticancer activity of crude methanol extract and sub-fractions were evaluated, using MTT assay. The induction of apoptosis was determined by analyzing DNA fragmentation in breast cancer cells treated with active fraction of crude methanol extract using agarose gel electrophoresis. To investigate molecular mechanism of apoptosis, gene expression levels of p53 and Bcl-2 were measured using quantitative real time PCR.

***Results: ***Fraction 10 was the most active fraction and was detected with HPLC as luteolin. The 50% cell cytotoxic concentration (CC_50_) of crude methanol extract and luteolin was 250 and 28 µg/ml, respectively. *This fraction was found to be an apoptotic agent against MDA-MB 231 cells, which leads to causing DNA fragmentation. *The mRNA expression level of Bcl-2 and p53 was significantly decreased and increased respectively in cancer cells treated by luteolin.

***Conclusion:*** *The* *results suggested that* Luteolin isolated from *Avicennia marina* could probably induce apoptosis on breast cancer cell line by the regulation of p53 and Bcl-2 pathways.

## Introduction

Breast cancer is the second most common cause of death among all forms of cancer in women, which has made the prevention and treatment of breast cancer a major health issue and healthcare goal (1). Currently, screening anticancer agents from natural products is a more effective way for drug discovery. About 25% of prescribed drugs in the world originate from plants and over 3000 species of plants have been reported to have anticancer properties (2, 3).

 The medicinal plant of Avicennia marina (Avicenniaceae family) is one of mangrove species tree native to Southern Africa (4). This plant grows along the coast of Persian Gulf, including Iran, and has been used widely in traditional medicine for the treatment of skin disease and rheumatoid (5).

Medicinal plants cause apoptotic process which is modulated by various tumor suppressor genes including p53. Apoptosis is a programmed cell death in response to a variety of stimuli and is usually characterized through distinct set of morphological hallmarks, including membrane blebbing, cytoplasmic and nuclear shrinkage, and nuclear DNA fragmentation in cells due to endonuclease activation. P53 is reported to induce apoptosis cell death by direct or indirect modulating expression of Bcl-2 family of proteins, Bcl-2 and Bax (6, 7). The Bcl-2 gene is an anti-apoptotic gene that represses initiation steps of apoptosis via inhibition of the pro-apoptotic proteins. Bcl-2 is over expressed in a high percentage of human breast cancer cells (8, 9).

Previous chemical investigation of A. marina leaf has shown the presence of flavonoids, iridoid glucosides, naphthoquinone derivatives, hydrocarbons and triterpenes (10, 11). Some investigations were done about antiviral, antimalarial, antibacterial and antifungal activities of A. marina (12). Previously anticancer activity of A. marina has been investigated against breast cancer cell line (13).

So far mechanism of anticancer activity of A. marina extract has not been investigated against cancer cell lines. Hence, the present study is focused on evaluating anticancer potentials of fractions against breast cancer cell line (MDA-MB 231) and human embryonic kidney normal cell line (HEK 293) through Bcl-2 and P53. 

## Materials and Methods

***Plant material and extraction of compounds***

Two Kg leaves of A. marina were collected from mangrove forests of Qeshm Island (Hormozgan, Iran), in December 2009. The voucher specimen was deposited in the Herbarium of Isfahan University (Iran). Leaves were carefully dried in a well-ventilated dark room and powdered. The plant material (50 g) was extracted with 500 ml of 90% MeOH at the room temperature (25–28°C) by maceration method (3×24 hr). The methanol extract was filtered and concentrated by a rotary evaporator (Steroglass, Italy) and then freeze dried (Zirbus, Germany).

***Isolation of compounds*** ***and high-performance liquid chromatography (HPLC) analysis***

Silica-gel column fractionation chromatography was carried out with the dried crude methanol extract (5 g) of A. marina eluted with chloroform: ethyl acetate (9:1 to 1:9, v/v).

 Fractions 1–14 (0.50, 0.38, 0.42, 0.39, 0.44, 0.35, 0.31, 0.30, 0.25, 0.28, 0.20, 0.40, 0.25 and 0.15 g) were obtained. The anticancer activity of 14 fractions was performed by MTT assay. Fraction 10 was the most active fraction and was detected with HPLC asluteolins.

HPLC screening of fraction 10 was carried out. HPLC was performed on a HITACHI Series HPLC system equipped with L-7100 pump and an L-7100 UV–vis detector. Peaks were separated on a RPC18 column using the mobile phase [methanol/acetone/water (70:20:10, v/v/v)]. The flow rate of the mobile phase was 1.5 ml min–1. The absorption of analytes was detected at 450 nm. Samples were injected to the HPLC bed manually with injection volume as 5 μl. T2000 software was used for peak integration and calculation. 

***Culture medium and cell lines***

MDA-MB 231 (human breast cancer) and HEK (normal human embryonic kidney) cell lines were acquired from National Cell Bank of Pasture Institute, Tehran, Iran. Cell lines were cultured in Dulbecco’s Modified Eagle Medium (DMEM) complemented with 10% heat-inactivated fetal bovine serum (FBS), 100 U ml^-1^ penicillin and 100 μg ml^-1^ streptomycin and 5 mM L- glutamine. The cell lines were grown at 37°C in a humidified atmosphere containing 5% CO2. All reagents and cell culture media were purchased from Gibco Company (Germany).

***Cytotoxicity assay***

The MTT [3-(4,5-dimethylthiazol-2-yl)-2,5-diphenyl-tetrazolium bromide] colorimetric assay was used to evaluate the antiproliferative activity of samples (methanolic crude extract and its fractions). This assay is based on the metabolic reduction of soluble MTT by mitochondrial enzyme activity of viable tumor cells, into an insoluble color formazan product, which can be measured spectrophotometrically after dissolving in dimethylsulfoxide (DMSO). Cellular toxicity of the crude methanol extracts from A. marina leaves on cultured cells was measured using this method (14). Briefly, 200 μl of cells (5×104 cells/ml) was seeded in 96 well microplates and incubated for 24 hr (37°C, 5% CO2 air humidified), then 20 μl of prepared concentrations of each sample was added. The samples were initially dissolved in DMSO before being added to the culture media. Control groups contained DMSO at the same concentration [0.5% (v/v)] of treated groups. After 48 hr of incubation, 20 μl of MTT solution (5 mg/ml in phosphate buffer solution) was added and the plates incubated for another 3 hr. 150 μl of medium containing MTT were then gently replaced by DMSO and pipetted to dissolve any formazan crystals formed. Absorbance was then determined at 560 nm by an ELISA plate reader (Awareness Technology Inc., stat fax 2100). Results were generated from three independent experiments; each experiment was performed in triplicate. Then 50% cell cytotoxic concentration (CC50) of A. marina was calculated via nonlinear regression of concentration-response curves.

***DNA fragmentation assay (apoptosis)***

The apoptosis induction in treated MDA-MB 231 cells was determined by measuring DNA fragmentation. The cells (2×10^6^ per ml) were incubated with fraction 10 and crude methanol extract at CC50 concentrations for 48 hr. Control cells were treated with 0.5% DMSO (v/v). After stimulation, the cells were washed twice with phosphate buffer solution (PBS). DNA was purified from the cells with high pure nucleic acid kit (Roche, USA) according to the standard protocol. Purified DNA was re-suspended in loading dye (Fermentas R0611) and run on 1.8% agarose gel in 1X TAE buffer. DNA fragmentations were visualized under UV transilluminator (Uvitec, England). 

***Determination of the expression levels of*** **apoptosis-regulatory genes**

The mRNA expression levels of two widely established apoptotic-related genes, Bcl-2 and p53 were analyzed using *two-step* RT-real time PCR assay as described (15, 16). Fraction 10 *at CC*_50 _*concentration* was used to stimulate the cells over the period of 2, 6 and 12 hr.

**Table 1:** The sequences of primers and probes used in real time PCR

Gene	Sequence
P53	Forward:5’- TAACAG TTCCTGCATGGGCGGC -3’ Reverse: 5’- AGG ACA GGC ACA AAC ACG CAC C -3’ Probe:5-(FAM)CGGAGG CCCATCCTCACCATCATC (MGB)-3
Bcl-2	Forward:5’-TTCGATCAGGAAGGCTAGAGTT-3’ Reverse:5’-TCGGTCTCCTAAAAGCAGGC-3’ Probe:5′-(FAM)CCCAGAGCATCAGGCCGCCAC(TAMRA)-3′
GAPDH	Forward:5’-CATGGGGAAGGTGAAGGTCGA-3’ Reverse: 5’-TTGGCTCCCCCCTGCAAATGAG-3’ Probe:5′-(JOE)CCGACTCTTGCCCTTCGAC(TAMRA)-3′

FAM; carboxy fluorescein amino-modified oligo. JOE; carboxy di chloro dimethoxy fluorescein amino-modified oligo. TAMRA; tetramethyl rhodamine amino-modified oligo, MGB: dihydrocyclopyrroloindole tripeptide minor groove binder

*RNA isolation and reverse transcription*

Total cellular RNA was isolated from the untreated and treated cells using the TriPure Isolation Reagent (Roche, USA), according to the manufacturer’s instructions. 5µg of RNA was reverse transcribed into cDNA in a reverse transcription reaction mixture containing 1x reaction buffer (Fermentas), 1 mM deoxy-nucleoside triphosphates (dNTPs) (Cinnagen DN7603C), 20 units of RNAase inhibitor (Fermentas E00381), 0.2 µg of random hexamer primer (Fermentas S0142), and 200 units of M-MuLV reverse transcriptase (Fermentas EP0441). After 10 min of incubation at room temperature to allow primer annealing, the reaction mixture was incubated 10 min at 25°C followed by 60 min at 42°C, terminated the reaction by heating at 70°C for 10 min heated to 95°C and chilled at 4°C for 5 min in a gradient thermal Cycler (Corbett Life Science). 5 µl of the resultant cDNA products was used as the template for PCR amplification.

*Quantitative real-time polymerase chain reaction assay for P53 and Bcl-2*

Real time PCR was performed to quantify the *expression levels of p53 and Bcl-2* in untreated and treated cells. A PCR reaction mixture of 50 μl containing 5 μl of dH_2_O, 25 μl of Taq Man, universal master mix, 5 μl of primer forward, 5 μl primer reverse, 5 μl FAM-BHQ1 probe and 5 μl *cDNA* were used. 

Three pairs of primers were separately used: two pairs to amplify the p53 and Bcl-2 genes, the other pair for the endogenous control gene, GAPDH *(Metabion International AG)* (Table 1). The probe and primer sets for each gene were designed using the OLIGO Primer Analysis Software (version 10, Molecular Biology Insights, Inc.). Real-time PCR was carried out on Light Cycler instrument (Roche Diagnostics). *The thermal* cycling conditions were as follows: 1 cycle denaturation of 95°C with 10 min hold, followed by 40 cycles of 95°C with 15 s hold, annealing temperature at 60°C (p53, Bcl2 and GAPDH) with a 60 sec hold. A negative control was included in each run to access specificity of primers and possible contamination. 

**Statistical analysis**

*SPSS 16.0 software was used to perform statistical analysis. T*hree independent experiments are presented as* the mean values ± standard deviation of the mean* (SD). *Analyze-of-variance (ANOVA) followed by LSD test (as the Post-Hoc) was used to assess* significance between the test sample and solvent control. *P-*value< 0.05 was considered to be statistically significant. The CC_50_ Values were calculated by Microsoft Excel 2003 Data.

## Results

***HPLC analysis***

**Figure 1:**
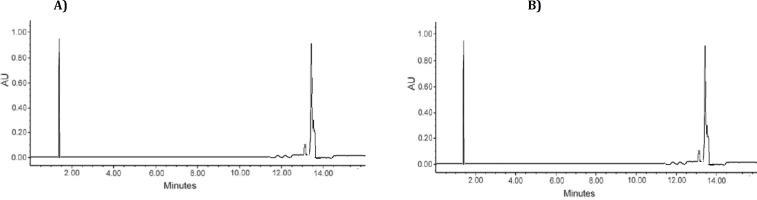
HPLC analysis of fraction 10 (A) and standard luteolin (B). Chromatographic conditions: RPC18 column, mobile phase: methanol/acetone/water = 70/20/10 (v/v/v) at 1.5 mL min^–1^

Figure 1A, 1B show the HPLC chromatograms of fraction 10 and standard luteolin. After purification, the major peak of fraction 10 was observed at a retention time of 13.5 min which was similar to luteolin standard peak. 

**Figure 2:**
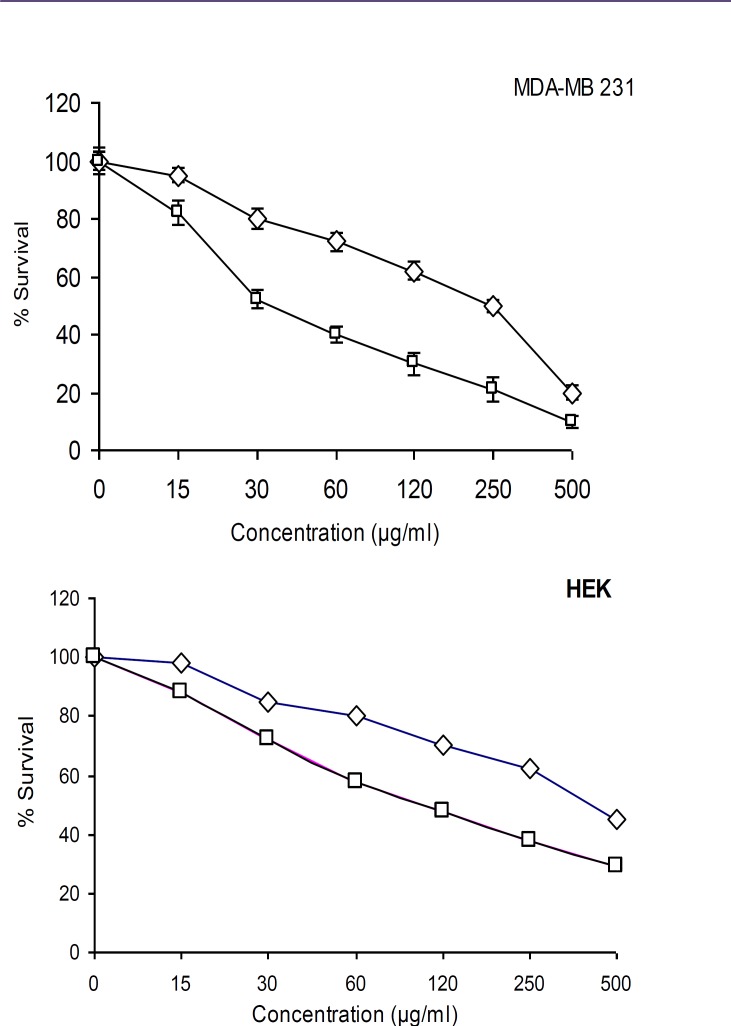
Cytotoxic activity of crude methanol extract ( ) and fraction 10 ( ) from *Avicennia* *marina* against MDA- MB 231 and HEK cell lines

***Cytotoxicity assay***

The crude methanol extract and fourteen fractions of *A. marina* were tested *for cytotoxicity against MDA- MB 231 and HEK 293.* Results showed that crude methanol extract and fraction 10 potentially inhibited viability of *MDA- MB 231 cell with* CC_50_ values of 250 and 28 *µg/ml,* *respectively.* Other fractions had low cytotoxic effect (data not shown). *Fraction 10 and crude methanol extract were tested under comparable conditions at different concentrations (500, 250, 125, 60, 30 and 15 µg/ml). As shown in *Figure 2*, these two extracts had significant dose-dependent inhibition on proliferation and viability of the MDA- MB 231 cancer cells and HEK normal cells. The results showed that cytotoxic activity of these extract on MDA- MB 231 cells was more active than HEK normal cells.*

**Induction of apoptosis**

**Figure 3:**
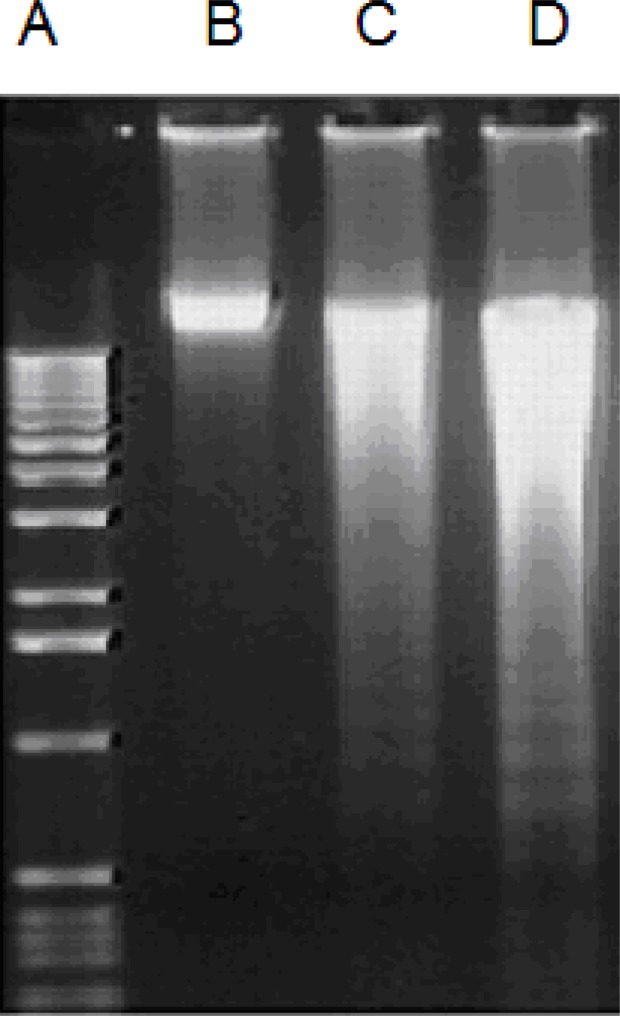
Agarose gel electrophoresis (1.8%) of the chromosomal DNA extracted of MDA-MB 231 cells. A: 1kb DNA marker, B: Control cells treated with 0.5 % DMSO, C and D: cells treated with fraction 10 and crude methanol extract respectively, at CC_50_ concentration for 48 hr

The cells treated with two extracts indicated the presence of DNA fragmentation which confirmed antiproliferative effect of extracts (Figure 3C and D). But control cells did not provide any fragmentation (Figure 3B). Breakdown of DNA molecule is one of the sign of inhibition of DNA replication due to *internucleosomal cleavage associated with apoptosis (*17*).*

***Expression level of apoptosis-related genes***

**Figure 4:**
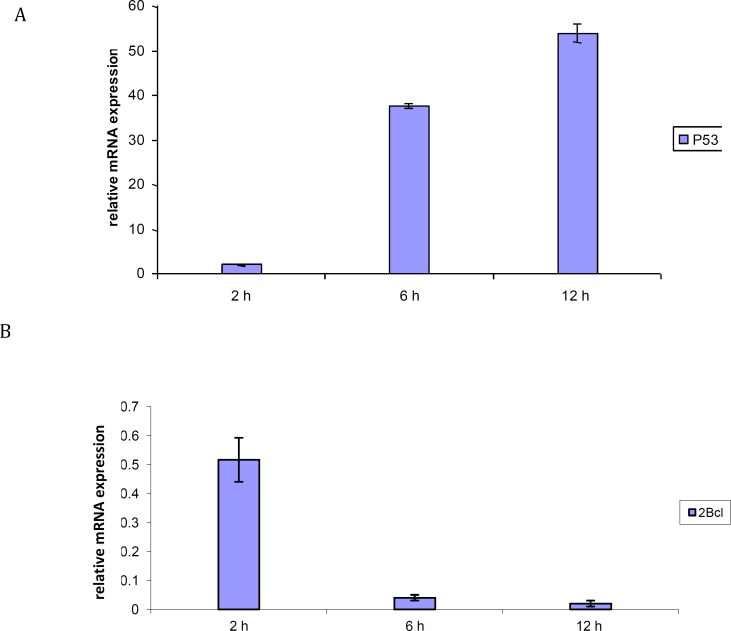
Time dependency effects of the p53 (A), Bcl-2 (B) mRNA levels in human breast cancer cell line, MDA- MB 231, incubated with 28 µg/ml of Luteolin after 2, 6 and 12 hr incubation. GAPDH was used as an endogenous control gene

The expression level of apoptosis-related genes in MDA- MB 231 cells which was induced by fraction 10 was determined. The mRNA levels of p53 and Bcl-2 were evaluated by real time PCR. Figure 4A showed that the expression level of p53 was increased 2, 37.5 and 54 folds after 2, 6 and 12 hr incubation with 28 µg/ml of luteolin, respectively. Figure 4B indicated that the expression level of Bcl-2 gene was decreased 0.5, 0.04 and 0.02 folds when the MDA- MB 231 cells were treated with 28 µg/ml of fraction 10 after 2, 6 and 12 hr incubation. Thus, the results indicate that the extract killed MDA- MB 231 cells through apoptosis mechanism mainly via these genes.

## Discussion

Medicinal plants have been used widely in traditional medicine for cancer treatment. In this study, anti-proliferative activity of different fractions of A. marina was tested and the results revealed that some fractions of this plant presented low activity while one fraction which contains only luteolin had high anti cancer activity. However according to the criteria of the American National Cancer Institute, the IC_50_ limit to consider an extract for further analysis is lower than 30 µg/ml (18). Thus, only fraction 10 “luteolin” extract could be considered as potential sources of anticancer compounds. Luteolin is a common flavonoid abundant in some plants such as *Artemisia afra, Cirsium maackii, Vitex negundo *and* Mentha longifolia* (19-22). Anti cancer activity of luteolin was investigated against MDA-MB 231 by expression levels of p53 and Bcl2. Previous studies have shown that luteolin stimulate induction of cancer cell apoptosis, cell cycle arrest, and anti-angiogenesis (23, 24). In this study, the mRNA expression levels of two apoptotic-related genes, p53 and Bcl-2 in MDA-MB 231 cells treated with the extract were investigated. Our results shown that luteolin induce apoptosis which is elicited through p53 and Bcl-2 genes. This finding is in agreement with many studies that demonstrated the role of p53 and Bcl-2 in inducing apoptosis (8, 25). P53 is a key tumor suppressor gene that has crucial function in apoptosis. Most of the drugs currently used to treat cancer patients exert their anti-tumor activity via p53-dependent tumor suppression. Thus, the development of effective drugs which can reactivate wild-type p53 tumor suppressor function is an attractive therapeutic strategy (26). The p53 can downregulate Bcl-2 which protects cells from apoptosis. Bcl-2 family members mediate anti-apoptotic signals in a wide variety of human cell systems (25, 27). Changa *et al* (2005) reported that luteolin has anti-cancer activity with IC50 between 7.2 to 32.59 µg/ml in immortalized human hepatoma cell line. The result revealed that luteolin can activate caspase-3 and decrease Bcl-XL (27). Previous studies have shown that some medicinal plants such as *Phyllanthus urinaria* can down regulate Bcl-2 expression which stimulates cytochrome c release from mitochondria (29).

## Conclusion

Based on the findings of the study, it can be concluded that A. marina leaves have an inhibitory effect on breast cancer cell lines. The results identified luteolin compound constitute as the most active compound in the crude methanol extract of A. marina.
